# ApoC-III is a novel inducer of calcification in human aortic valves

**DOI:** 10.1074/jbc.RA120.015700

**Published:** 2021-01-06

**Authors:** Florian Schlotter, Renata C.C. de Freitas, Maximillian A. Rogers, Mark C. Blaser, Pin-Jou Wu, Hideyuki Higashi, Arda Halu, Farwah Iqbal, Allison B. Andraski, Cayla N. Rodia, Shiori Kuraoka, Jennifer R. Wen, Michael Creager, Tan Pham, Joshua D. Hutcheson, Simon C. Body, Alison B. Kohan, Frank M. Sacks, Masanori Aikawa, Sasha A. Singh, Elena Aikawa

**Affiliations:** 1 Division of Cardiovascular Medicine, Department of Medicine, Center for Interdisciplinary Cardiovascular Sciences, Brigham and Women's Hospital, Harvard Medical School, Boston, Massachusetts, USA; 2 Channing Division of Network Medicine, Brigham and Women’s Hospital, Harvard Medical School, Boston, Massachusetts, USA; 3 Division of Cardiovascular Medicine, Department of Medicine, Center for Excellence in Vascular Biology, Brigham and Women's Hospital, Harvard Medical School, Boston, Massachusetts, USA; 4 Department of Nutrition and Department of Molecular Metabolism, Harvard T.H. Chan School of Public Health, Boston, Massachusetts, USA; 5 Department of Nutritional Sciences, University of Connecticut, Storrs, Connecticut, USA; 6 Department of Biomedical Engineering, Florida International University, Miami, Florida, USA; 7 Department of Anesthesiology, Boston University School of Medicine, Boston, Massachusetts, USA; 8 Division of Endocrinology and Metabolism, Department of Medicine, University of Pittsburgh, Pittsburgh, Pennsylvania, USA; 9 Department of Human Pathology, Sechenov First Moscow State Medical University, Moscow, Russia

**Keywords:** calcific aortic valve disease, valvular interstitial cells, fibrosis, lipids, apoC-III, αSMA, α-smooth muscle actin, apoB, apolipoprotein B, AS, aortic stenosis, AV, aortic valve, BMP, bone morphogenic protein, C, calcific, CAVD, calcific aortic valve disease, DDA, data-dependent acquisition, DMEM, Dulbecco's modified Eagle's medium, F, fibrotic, FACS, fluorescence-activated cell sorting, FBS, fetal bovine serum, FDR, false discovery rate, HDL, high-density lipoprotein, IL-6, interleukin-6, LDL-C, low-density lipoprotein cholesterol, lp(a), lipoprotein(a), NC, noncalcific, NF, nonfibrotic, NM, normal media, PM, procalcifying media, PPI, protein–protein interaction, PRM, parallel reaction monitoring, RT, retention time, VICs, valvular interstitial cells

## Abstract

Calcific aortic valve disease (CAVD) occurs when subpopulations of valve cells undergo specific differentiation pathways, promoting tissue fibrosis and calcification. Lipoprotein particles carry oxidized lipids that promote valvular disease, but low-density lipoprotein–lowering therapies have failed in clinical trials, and there are currently no pharmacological interventions available for this disease. Apolipoproteins are known promoters of atherosclerosis, but whether they possess pathogenic properties in CAVD is less clear. To search for a possible link, we assessed 12 apolipoproteins in nonfibrotic/noncalcific and fibrotic/calcific aortic valve tissues by proteomics and immunohistochemistry to understand if they were enriched in calcified areas. Eight apolipoproteins (apoA-I, apoA-II, apoA-IV, apoB, apoC-III, apoD, apoL-I, and apoM) were enriched in the calcific *versus* nonfibrotic/noncalcific tissues. Apo(a), apoB, apoC-III, apoE, and apoJ localized within the disease-prone fibrosa and colocalized with calcific regions as detected by immunohistochemistry. Circulating apoC-III on lipoprotein(a) is a potential biomarker of aortic stenosis incidence and progression, but whether apoC-III also induces aortic valve calcification is unknown. We found that apoC-III was increased in fibrotic and calcific tissues and observed within the calcification-prone fibrosa layer as well as around calcification. In addition, we showed that apoC-III induced calcification in primary human valvular cell cultures *via* a mitochondrial dysfunction/inflammation-mediated pathway. This study provides a first assessment of a broad array of apolipoproteins in CAVD tissues, demonstrates that specific apolipoproteins associate with valvular calcification, and implicates apoC-III as an active and modifiable driver of CAVD beyond its potential role as a biomarker.

Calcific aortic valve disease (CAVD) is a chronic disorder with increasing prevalence in the Western world but lacks pharmacological therapies. Valvular interstitial cells (VICs) maintain a quiescent fibroblast phenotype in healthy heart valves (1), which when challenged by pathologic stimuli, undergo myofibroblastic or osteoblastic differentiation, promoting tissue fibrosis and calcification leading to CAVD and aortic stenosis (AS) (1). Multiple aspects that regulate these processes have been identified, with lipoproteins and associated proinflammatory factors (2) suggested to play a major role in CAVD pathogenesis. However, the assessment of the role of apolipoproteins and proteins involved in the formation of lipoproteins, in CAVD, remains scant.

Apolipoproteins form a special class of proteins that can associate with lipids, serve as essential structural and functional components in lipoproteins, and have well-established roles in regulating atherosclerosis (3). The knowledge of specific effects of apolipoproteins on aortic valve (AV) disease pathogenesis is largely limited to studies on apolipoprotein B (apoB)–containing lipoproteins that serve as carriers for oxidized phospholipids (4), angiotensin-converting enzyme (5), as well as lipoprotein(a) [lp(a)] that carries proinflammatory lipids and promotes CAVD (2). Genetically, *LPA* is the only lipoprotein gene that has been associated with AV calcification (6). At the epidemiological level, apoB (7), lp(a) (8, 9), and apoC-III (10, 11) are associated with CAVD, and apoA-I has demonstrated an inverse relationship to risk for AS incidence (12) and its hemodynamic progression (13). Low-density lipoprotein cholesterol (LDL-C), oxidized phospholipids, and triglycerides are associated with onset of CAVD (10, 14, 15) and are introduced into the valvular matrix as components of lipoproteins. In a large Mendelian randomization study, plasma LDL-C was related to the presence of AV calcium, and a LDL-C genetic risk score was associated with increased incidence of AS (16). *In vitro* evidence suggests that autotaxin derived from circulating lp(a) particles promotes inflammation and mineralization of AV (2) and participates in the metabolism of oxidized phospholipids that induce osteogenic changes in VICs (5). Oxidized high-density lipoprotein (HDL) promotes VIC calcification (17). *In vivo*, apolipoprotein E–deficient and LDL-receptor deficient mice, and LDL receptor–deficient apo B-100 (Ldlr^−/−^Apob100/100) mice are utilized to model CAVD. In addition, injection of an apoA-I mimetic peptide to rabbits and mice led to a reduction in fibrosis and calcification (14, 18, 19, 20). Each of these hyperlipidemic models represents aspects of CAVD pathophysiology to varying degrees (21). Despite these lipid metabolism links to CAVD, randomized controlled trials testing pharmacological LDL lowering with statins have failed to demonstrate beneficial effects on CAVD progression (22, 23, 24). While apolipoproteins and apolipoprotein-associated molecules have been implicated in various studies of CAVD, there has not been an unbiased systematic study of apolipoprotein prevalence in human CAVD, and mechanistic roles of apolipoproteins in CAVD are poorly defined.

Here, we sought to assess whether apolipoproteins contribute to CAVD pathogenesis by quantitatively mapping apolipoproteins across CAVD tissues that represent disease progression and evaluating calcification effects of select commercially available purified human apolipoproteins as an *in vitro* proof of concept.

## Results

### Apolipoproteins associate with CAVD severity

**Figure 1:**
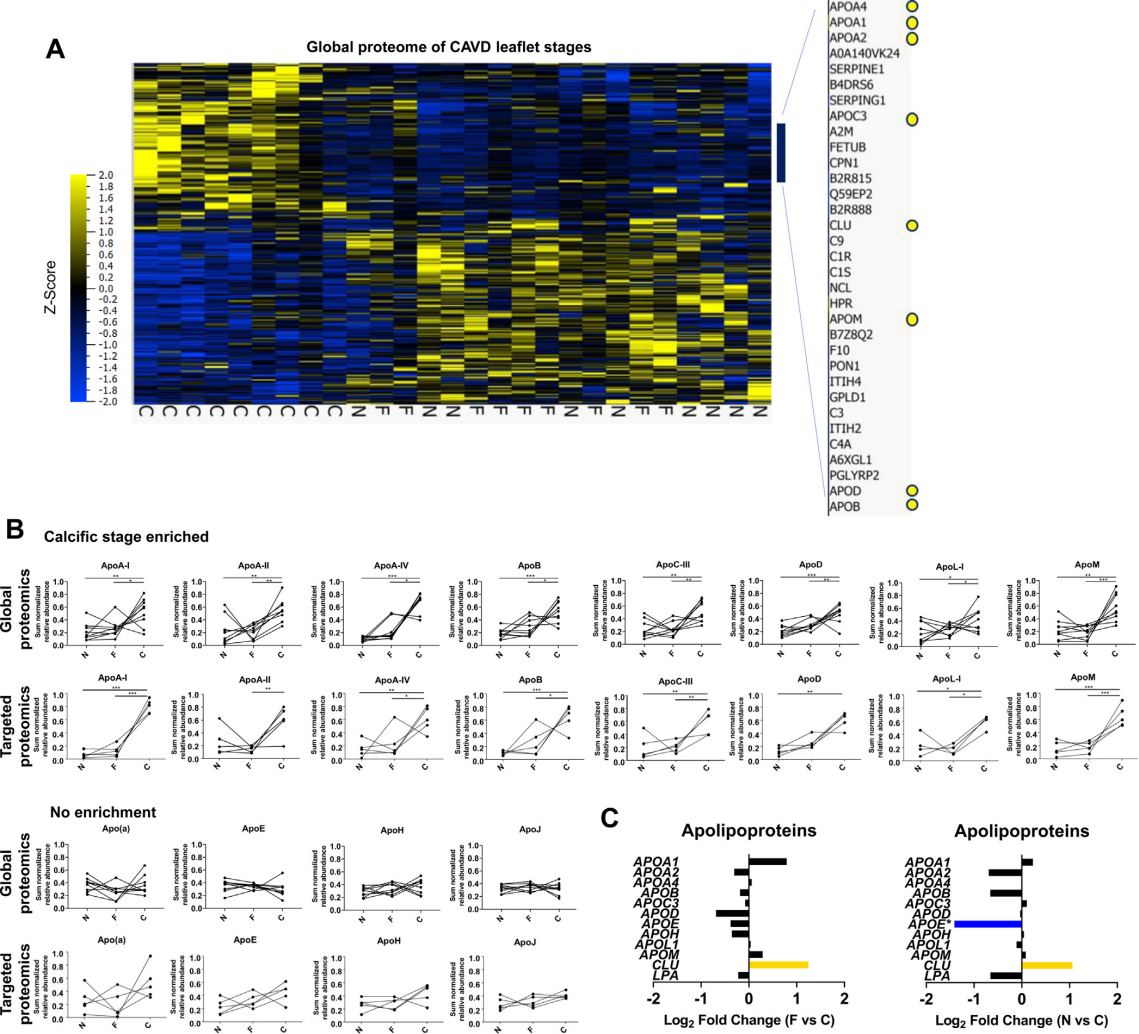
**Specific apolipoproteins predominate in calcific aortic valve disease (CAVD) tissues.***A*, hierarchical cluster analysis (multigroup comparison; *q* ≤ 0.3) of the CAVD disease stage proteome (*n* = 9 donors; *N* = 1238 proteins with three or more unique peptides). *Inset*, is a zoomed view of apolipoproteins enrichment in CAVD tissue. *B*, sum-normalized apolipoprotein abundances across CAVD stages from the global proteomics data (*upper panel*; *n* = 9 donors) and from the parallel-reaction monitoring data (*lower panel*; *n* = 5 donors; averaged sum-normalized signal of one to three peptides per donor). *C*, fold change of gene expression of apolipoproteins between fibrotic and calcific or nonfibrotic/noncalcific and CAVD tissues from RNA-Seq. ∗*p* < 0.05, ∗∗*p* < 0.01, ∗∗∗*p* < 0.001, one-way ANOVA with Bonferroni multiple comparison test or Kruskal–Wallis test with Dunn's multiple comparison test for nonparametric data. C, calcific tissue; F, fibrotic tissue; N, nonfibrotic/noncalcific tissue.

**Figure 2:**
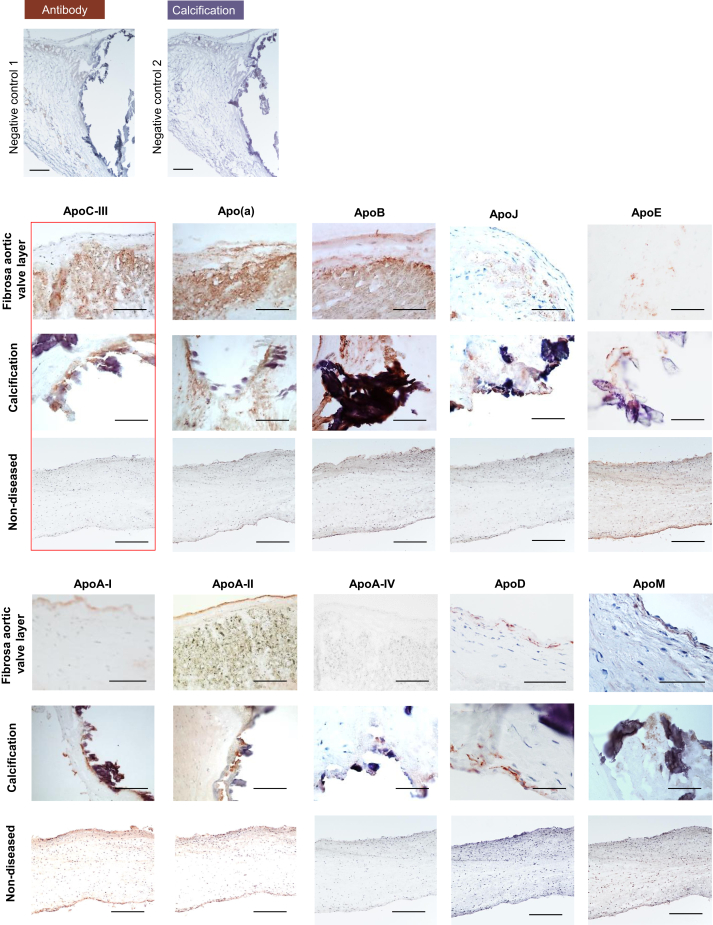
**Aortic valve apolipoprotein localization and association with calcification.** Negative control 1 and 2: staining without primary antibody, but horseradish peroxidase–conjugated antimouse and anti-rabbit secondary antibodies; representative immunohistochemistry images for apolipoproteins on human aortic valve tissue in the fibrosa layer (*top panel*, fibrosa facing up), around calcification (*middle panel*), and on nondiseased controls. The scale bar represents 100 μm.

To characterize the human AV apolipoprotein distribution across different CAVD tissues, we leveraged the availability of CAVD stage proteomics data (25) to focus our analysis on apolipoproteins. These segments were defined by three major CAVD tissues: nonfibrotic/noncalcific (NF/NC), fibrotic (F), and calcific (C). We noted the prevalence of several apolipoproteins in calcific CAVD tissue (Fig. 1*A*), including several classical apolipoproteins, such as apoA-I, apoA-II, apoC-III, and apoB (Fig. 1*A*). We extracted all classical apolipoproteins (26) from the global proteome data set (Table S1) and compared their abundance profiles using their sum-normalized distributions (Fig. 1*B*). Two phenomena were observed: (1) eight apolipoproteins (apoA-I, apoA-II, apoA-IV, apoB, apoC-III, apoD, apoL-I, and apoM) were enriched in the calcific CAVD tissue relative to the NF/NC, and some also relative to the fibrotic CAVD tissue (Fig. 1*B*) and (2) four apolipoproteins (apo(a), apoE, apoH, and apoJ) displayed no significant alterations across CAVD tissues (Fig. 1*B*).

We followed up on these findings by employing a targeted MS application of parallel reaction monitoring (PRM) to quantify the relative abundances of between one to three peptides for these 12 apolipoproteins identified in the global proteome in AV samples from five donors. We first validated a previously established peptide library (25, 27) on human HDL size fractions (27) (Supporting Information 1), examined apolipoproteins in AVs from the five CAVD donors, and verified the relative distributions demonstrated by our global proteomics analysis (Fig. 1*B*). We also reanalyzed global RNA-Seq data from three CAVD donor leaflets (25) to specifically determine whether prevalence of apolipoproteins in calcific CAVD tissue could be due to altered levels in local transcription; however, while some apolipoprotein genes tended to increase in the calcific CAVD tissue, only apoE was significantly changed (Fig. 1*C*). The gene that encodes apoJ (*CLU*) was significantly decreased in calcific CAVD tissue (Fig. 1*C*). This disconnect between valvular transcriptomics and proteomics quantification of apolipoproteins is consistent with global trends that we demonstrated previously (25).

### Apolipoproteins are deposited within extracellular matrix and colocalize with calcification

In addition to our CAVD tissue proteomics analysis examination of relative abundances of apolipoproteins across disease progression, we used immunohistochemistry to identify the spatial localization of apolipoproteins throughout the AV interstitium and their association with extracellular matrix and calcification. ApoC-III, apo(a), apoB, and to a lesser extent apoJ and apoE exhibited a prominent and diffuse signal in collagen-enriched matrix of the disease-prone fibrosa layer (Fig. 2, *fibrosa panel*). In addition, these apolipoproteins were highly abundant around calcific regions (Fig. 2, *calcification panel*). Of note, the tissue distribution of apo(a) and apoJ appeared more diffuse and not strictly limited to calcification. ApoA-I, apoA-II, apoA-IV, apoD, and apoM colocalized with calcification deposits in the fibrosa layer but were not enriched in the noncalcified extracellular matrix of the fibrosa layer.

**Figure 3:**
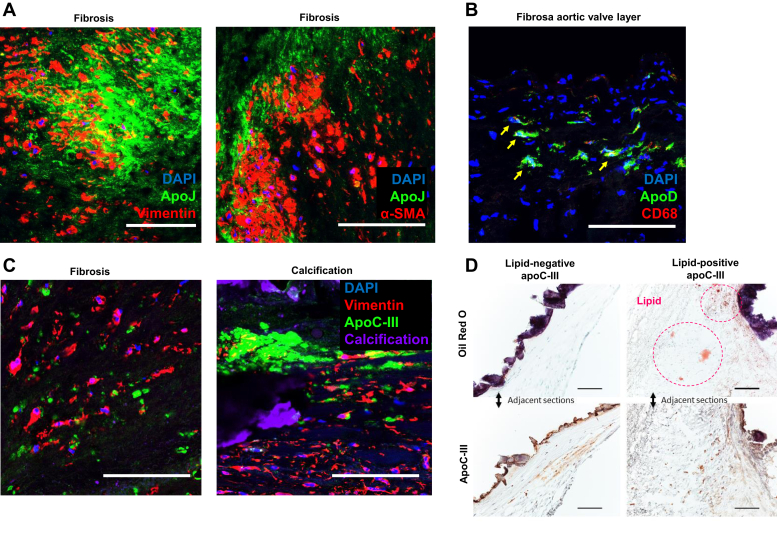
**Tissue apolipoprotein distribution.***A*, coimmunofluorescence for apoJ and vimentin (*left panel*) and apoJ and αSMA (*right panel*). *B*, coimmunofluorescence for apoD and CD68; *yellow arrows* indicate double-positive cells. The scale bar represents 100 μm. Observations were confirmed in three independent donors. *C*, immunofluorescence for apoC-III, vimentin (valvular interstitial cells), and calcification (Osteosense 680; near-infrared calcium tracer [Perkin Elmer, USA]) on fibrotic and calcific human aortic valve sections. *D*, oil red O staining of calcific human aortic valve tissue and apoC-III immunohistochemistry on serial sections identifying areas of lipid-poor apoC-III. αSMA, α-smooth muscle actin; DAPI, 4′,6-diamidino-2-phenylindole.

ApoJ immunofluorescence demonstrated both cellular and extracellular expression patterns (Fig. 3*A*), particularly in collagen-rich fibrosa, with high immunofluorescence signal in and around α-smooth muscle actin (αSMA)-positive cells and vimentin-positive cells, suggesting heightened expression by myofibroblast-like VICs (Fig. 3*A*). While lipid-containing macrophage foam cells were largely lacking within diseased AV portions, immunoreactive apoD colocalized with a limited number of CD68-positive macrophages (Fig. 3*B*). Fluorescence-activated cell sorting (FACS) corroborated these findings. Freshly isolated from a whole aortic leaflet, noncultured cells were gated for live cells, and only single cells were selected for analysis. We identified an αSMA-positive population (2.5% of cells), of which 80% of cells were double positive for apoJ and αSMA. Approximately, 3% of cells were positive for CD68 with 80% double positive for CD68 and apoD (Supporting Information 2 for all FACS analyses).

### ApoC-III contributes to AV calcification

Clinical evidence indicates that apoC-III may promote cardiovascular events independent of traditional proatherogenic lipoproteins and lipids such as LDL or triglycerides (17). ApoC-III is currently under investigation in clinical trials as a therapeutic target for dyslipidemia (18, 22). In light of these findings and our observations that apoC-III was significantly enriched in calcific CAVD tissue, highly present in the disease-prone fibrosa layer, and abundant around calcific nodules, we focused our investigation on whether apoC-III can promote AV calcification as an *in vitro* proof of concept of the observations we made with our proteomics and immunohistochemistry-generated apolipoprotein CAVD atlas.

**Figure 4:**
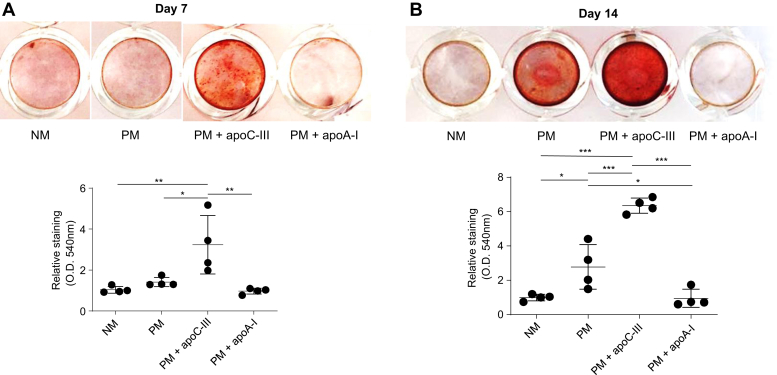
**ApoC-III is a contributor to valvular interstitial cell calcification.***A*, representative alizarin red staining of valvular interstitial cells incubated with normal media (NM), procalcifying media (PM), PM with 100 μg/ml human apoC-III (PM + apoC-III), and PM with 100 μg/ml human apoA-I (PM + apoA-I) at day 7 after first incubation; and alizarin red absorbance quantification (*n* = 4 independent donors, all passage 2). *B*, representative alizarin red staining of valvular interstitial cells incubated with NM, PM, PM + apoC-III, and PM + apoA-I at day 14 after first incubation; and alizarin red absorbance quantification (*n* = 4 independent donors, all passage 2).

Immunofluorescence specifically identified apoC-III in fibrotic and calcific AV regions (Fig. 3*C*), corroborating our histopathology data (Fig. 2*A*). In addition, apoC-III was found in both lipid-rich and lipid-poor areas around calcific nodules of human AV leaflets (oil red O-positive and -negative, respectively; Fig. 3*D*), indicating that apoC-III may contribute to AV calcification, independent of its function in lipoprotein metabolism. In order to confirm that apoC-III protein alone can promote calcification, we incubated primary human VICs with human apoC-III and measured calcification at multiple time points using an established *in vitro* calcification assay (28). Calcific burden was significantly elevated in comparison to non–apoC-III-incubated controls at both incubation time points, demonstrating apoC-III-driven induction of VIC calcification (Fig. 4, *A*–*B*). Notably, apoA-I incubation did not increase calcification (Fig. 4, *A*–*B*), implying that this was a specific effect of apoC-III protein.

### ApoC-III promotes mitochondrial stress and inflammation-mediated calcification

**Figure 5:**
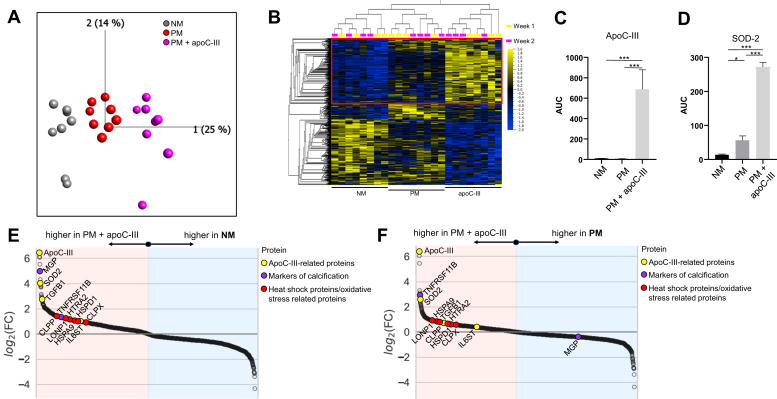
**ApoC-III drives mitochondrial dysfunction and oxidative stress.***A*, principal component analysis of 2773 proteins with ≥2 unique peptides. *B*, heat map of 1365 proteins with *q* ≤ 0.1 between normal medium (NM), procalcifying medium (PM), and PM + apoC-III. *Red box* highlights enriched proteins in PM + apoC-III *versus* NM and PM. *C*–*D*, apoC-III and superoxide dismutase 2 (SOD-2) are significantly enriched in PM + apoC-III quantified by MS, respectively. *E*, differentially enriched proteins in NM *versus* PM + apoC-III. *F*, differentially enriched proteins in PM *versus* PM + apoC-III. *n* = 4 donors, day 7 and day 14 data.

To examine mechanisms by which apoC-III stimulates VIC calcification, we investigated the *in vitro* proteome of apoC-III-incubated VICs. Principal component analysis suggested a major impact of the media incubation on the VIC proteome based on a total of 2773 identified proteins (Fig. 5*A* and Table S2). After FDR filtering for proteins with a *q*-value ≤0.1 between our three media conditions, 1365 proteins remained (Fig. 5*B*). Of note, MS readily detected the exogenous apoC-III incubation (Fig. 5*C*). Superoxide dismutase 2, a protein involved in clearance of mitochondrial reactive oxygen species, was one of the most prominently upregulated proteins after apoC-III incubation relative to normal media (NM) and procalcifying media (PM) and may signify mitochondrial dysfunction in apoC-III incubated VICs (Fig. 5*D*). Besides superoxide dismutase 2, apoC-III increased several mitochondrial and oxidative stress–related proteins (HSPA9, HSPD1, HTRA2, LON1, CLPP, and CLPX) and elements of inflammation cascades (TGFB1 and IL6ST) relative to both NM and PM (Fig. 5, *E*–*F*, respectively).

**Figure 6:**
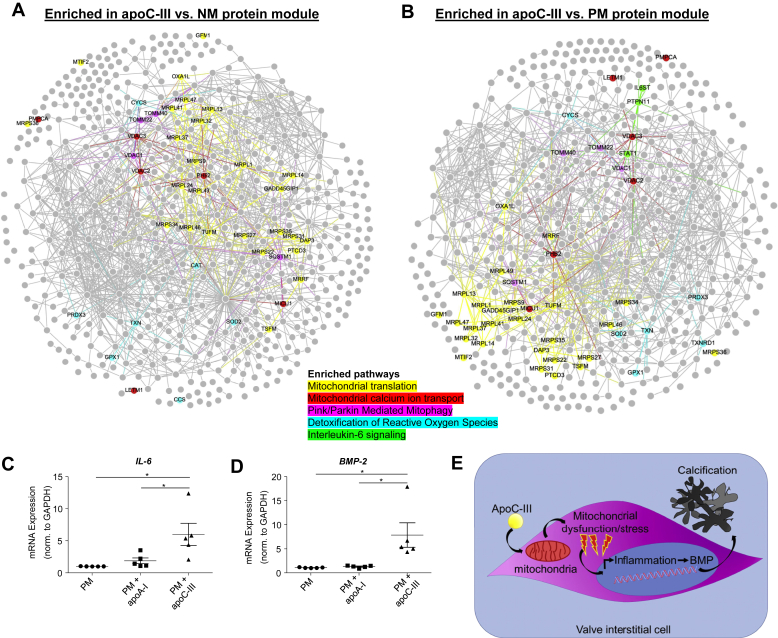
**ApoC-III may act through mitochondrial stress–mediated inflammation.***A*, proteins with *q* ≤ 0.1 between normal medium (NM), procalcifying medium (PM), and PM + apoC-III increased in PM + apoC-III *versus* NM with proteins of major pathways highlighted (*n* = 4 donors, day 7 and day 14 data). *B*, proteins with *q* ≤ 0.1 between NM, PM, and PM + apoC-III increased in PM + apoC-III *versus* PM with proteins of major pathways highlighted (*n* = 4 donors, day 7 and day 14 data). *C*, ApoC-III incubation increased interleukin-6 (IL-6) mRNA expression (*n* = 5 independent donors, apoC-III or apoA-I incubation for 48 h). *D*, bone morphogenetic protein 2 (BMP-2) mRNA was highly expressed in valvular interstitial cells incubated with 100 μg/ml apoC-III (*n* = 5 independent donors, apoC-III or apoA-I incubation for 48 h). Data are expressed as mean ± SEM. ∗*p* < 0.05, ∗∗*p* < 0.01, ∗∗∗*p* < 0.001, one-way ANOVA with Bonferroni multiple comparison test or Kruskal–Wallis test with Dunn's correction. *E*, potential role of ApoC-III in valvular calcification. ApoC-III induces mitochondrial dysfunction–driven inflammation that promotes calcification-associated processes.

We next investigated differentially enriched protein modules for enriched proteins after apoC-III incubation relative to NM and PM and detected a significant enrichment in mitochondrial, mitophagy, and reactive oxygen species–related pathways (Fig. 6, *A*–*B*, respectively; complete modules in Supporting Information 3; all significantly enriched pathways per media in Supporting Information 4, *A*–*B*). In addition, the identified apoC-III–related pathways had significant connections with previously defined inflammation (29), calcification (29), and CAVD (25) modules on the protein–protein interaction (PPI) and pathway levels (Supporting Information 5). Mitochondrial dysfunction and ensuing oxidative stress may elevate inflammation (4). The interleukin-6 (IL-6) pathway was significantly enriched after apoC-III incubation. In agreement with identification of the IL-6 pathway by proteomics and pathway analysis, IL-6 gene expression was significantly elevated after apoC-III incubation (Fig. 6*C*). Inflammation-related pathways have been shown to potentiate VIC calcification (2); therefore, we assessed a possibility that apoC-III may drive calcification potential *via* inflammation-mediated bone morphogenic protein (BMP) expression. ApoC-III incubation increased BMP-2 expression in human VICs (Fig. 6*D*), supporting an involvement of a mitochondrial dysfunction/inflammation-mediated calcification pathway triggered by apoC-III in human VICs (Fig. 6*E*).

## Discussion

In this study, we demonstrated the following in the CAVD: (1) apolipoproteins exhibit differential abundances across stages of disease pathogenesis through high-precision targeted proteomics; (2) apolipoprotein tissue localization reveals preferential diffuse deposition in acellular extracellular matrix of a subset of pathogenic apolipoproteins; (3) apoC-III promotes calcification in cultured primary human VICs; and (4) apoC-III induced a mitochondrial dysfunction/inflammation-mediated calcification pathway.

We previously indicated the value of subsectioning AV leaflets for molecular profiling across disease progression (25). Although harvested from calcified valves, the inhomogeneous disease progression allowed us to identify NF and NC portions as well as the CAVD spectrum of fibrotic and calcific tissues from the same donor (confirmed by histopathology), thereby reducing interdonor variability. We leveraged the power of this approach to explore the differential distribution of the apolipoproteome. In the present study, our use of quantitative targeted proteomics integrated with the spatial immunohistochemical analysis allowed us to illustrate the presence of a broad range of apolipoproteins present in CAVD tissues and tissue layers. Our findings further suggest the presence of numerous apolipoproteins in pathologically remodeled AV tissue that is not limited to the atherogenic lipoproteins Lp(a), LDL, and very low-density lipoprotein; as well as their major composites apo(a), apoB, and apoC-III, respectively. Differentially localized apolipoproteins in AV leaflets support growing evidence suggesting that a variety of apolipoproteins may play unique roles in disease initiation and progression, either through direct pathological interaction with cells and surrounding matrix or through their cargo function including their lipid-carrying properties. It is well established that damage to valvular endothelium serves as a first insult to enable infiltration and retention of blood-derived factors into the valvular interstitial space. Exposure of VICs to these factors subsequently drives disease development through cellular stress and pathologic differentiation. The role of apolipoproteins on these processes in VICs remains insufficiently elucidated. Our finding of an abundance of select apolipoproteins retained within the extracellular matrix and the paucity of foam-like macrophages in calcific AV tissue points toward the lack of a macrophage-driven apolipoprotein and lipid removal mechanism in CAVD. In addition, our data show that local transcription of the apolipoprotein genes fails to account for the large differences in apolipoprotein abundance, suggesting that plasma-derived apolipoprotein infiltration likely explains much of this pathological effect.

In the light of the expanding body of literature on Lp(a) and CAVD, the absence of an association between apo(a) by targeted proteomics and disease severity in this study was unexpected but can be easily explained by methodology limitations. An apo(a)-specific peptide that we were able to detect (GTYSTTVTGR) is part of the apo(a) kringle domain replicates, which are present in highly variable numbers among individuals (23), and may thus not be suitable for absolute PRM quantification. Although, trends within a given donor tissue would still be valid, interdonor comparisons are challenging. To date, no mass spectrum standard exists for apo(a), in large part because of the confounding features highlighted previously. It has also been suggested that Lp(a) accumulates at sites of wound healing secondary to its binding capacity to fibrin, thus accumulating at sites of minor injury in the very early stage of AV lesion initiation (30), thus impacting the quantification in our sectioning approach.

To date, a limited number of apolipoproteins have been shown to play a role in the pathogenesis of CAVD, mostly apo(a) (31) and apoB (5). We selected two commercially available purified human apolipoproteins, which associated with disease progression in our tissue proteomics data set and represent large structural components of the major lipoprotein classes of HDL and very low-density lipoprotein, respectively. In addition to demonstrating that apoC-III enhances VIC calcification in concentrations observed in patient plasma (4), we found that apoC-III likely mediates valvular calcification *via* a mitochondrial dysfunction–driven inflammatory mechanism including an IL-6/BMP-2 pathway, a signaling cascade previously demonstrated as a major promoter of VIC calcification (2, 12). Moreover, network analysis revealed connections of the apoC-III–related pathways with previously identified inflammation (29), calcification (29), and CAVD-specific (25) protein and pathway networks. Our results are in line with prior findings, suggesting that ApoC-III drives proinflammatory signaling cascades (32, 33). Our novel findings are also in agreement with epidemiological data of an association between circulating plasma apoC-III levels and AV sclerosis (10)—a valvular pathology that typically occurs prior to the onset of valvular calcification. However, our data provide a novel mechanistic link of apoC-III with valvular calcification beyond an epidemiological association.

ApoA-I served as a second commercially available purified human apolipoprotein that we investigated for effects on calcification. The absence of *in vitro* calcification induction is consistent with prior findings, where apoA-I had an inverse relationship to risk for AS incidence (12) and hemodynamic AS progression (13). Furthermore, *in vivo* data suggested that injection of an apoA-I mimetic peptide to rabbits and mice led to a reduction in fibrosis and calcification (14, 18, 19, 20). ApoA-I was shown to induce osteoprotegerin secretion and reduce tumor necrosis factor-α mRNA expression in VICs (19), which may counteract pathological VIC differentiation. However, this is the first time to our knowledge, that apoA-I calcification effects are documented *in vitro*, notably in primary human valvular interstitial cells.

Our study supports the need for further research on a broad range of apolipoproteins in CAVD pathobiology and a need to assess whether targeted lowering of specific apolipoproteins, including in ongoing clinical apoC-III-trials not being investigated for CAVD also provide therapeutic CAVD benefits.

## Conclusions

Our results provide a global mapping of human valvular apolipoproteins, open new avenues to study the interplay between apolipoproteins and CAVD, and implicate apoC-III as an active and modifiable contributor to human valvular calcification.

## Experimental procedures

### AV dissection and proteolysis

AV leaflets were obtained from valve replacement surgeries for AV stenosis performed at Brigham and Women's Hospital with written informed consent provided by the patients (total 16 valve donors; Brigham and Women's Hospital Institutional Review Board protocol number: 2011P001703) (25). All procedures performed in studies involving human participants were in accordance with the 1964 Helsinki Declaration and its later amendments. Patients or the public were not involved in the design, or conduct, or reporting, or dissemination plans of our research. AV tissue dissection and proteolysis steps were established previously (25). In brief, the AV samples were immediately transferred from the operating room in Dulbecco's modified Eagle's medium (DMEM; Lonza, Switzerland) and snap frozen within 30 min. All samples were washed in cold PBS three times. We segmented each AV leaflet into the three stages reflecting NF/NC, fibrotic, (F), and calcific (C) as recently described by our group using molecular imaging (25). Transition zones were excluded from all analyses. The tissue samples were snap frozen in liquid nitrogen and pulverized with a hammer. The resulting tissue powder was resuspended in 100 μl radioimmunoprecipitation assay buffer (Thermo Fisher Scientific, USA) supplemented with 1% protease inhibitor cocktail (Roche, Switzerland). The samples were sonicated to shear the DNA and homogenize the tissue (Branson Sonifier 450, USA). We performed protein precipitation by adding 1 ml of 2:1 chloroform:methanol to each 100 μl of sample. Protein content was quantified using BCA kit (Thermo Fisher Scientific, USA). For proteolysis, we used the trypsin (Promega, USA) plus RapiGest (Waters, USA) strategy using 15 μg of protein per sample. The resulting tryptic peptide solutions were desalted by Oasis Hlb 1 cc (10 mg) columns (Waters, USA), dried with a tabletop speed vacuum (Thermo Fisher Scientific, USA), and resuspended in 40 μl of 5% acetonitrile (Optima LC–MS grade; Thermo Fisher Scientific, USA) and 5% formic acid (Sigma–Aldrich, USA) prepared in Optima LC–MS grade water (Thermo Fisher Scientific) for subsequent analysis by PRM MS.

### VIC isolation and cell culture

Endothelial cells were removed from human AVs by scratching the surface with a razor blade. VICs were then isolated using 1% collagenase (Sigma–Aldrich, USA) (2, 25, 28). The cells were expanded in growth media (DMEM [Lonza, Switzerland]) supplemented with 10% fetal bovine serum (FBS) (VWR, USA) and 1% streptomycin/penicillin (Corning, USA) until they reached 90% confluency. VICs at passages between two and four were used.

### *In vitro* VIC calcification and proteomics

VICs plated in 48-well plates (Corning, USA) were cultured in control NM containing DMEM with 5% FBS or PM containing DMEM with 5% FBS, 2 mmol/l monosodium phosphate (Sigma–Aldrich) (pH 7.4), and 50 μg/ml L-ascorbic acid. VICs were incubated with commercially available purified human apoC-III (ultrapure human apoCIII; Academy Biomedical Company, Inc, USA) or apoA-I (ultrapure human apoAI; Academy Biomedical Company, Inc, USA). Endotoxin levels were assessed (ToxinSensorTM Chromogenic LAL Endotoxin Assay Kit; GenScript, NJ, USA) and did not differ between apoA-I and apoC-III. For the calcification assay and VIC proteomics analyses, VICs were incubated for 7 and 14 days.

For the calcification assays, cells were then fixed with 10% formalin for 15 min. Matrix calcium deposition was analyzed by alizarin red staining (Lifeline Cell Technology, USA). Fixed cells were washed twice with distilled water and stained with 2% alizarin red for 15 min. Excess stain was washed twice with distilled water. Alizarin red staining was quantified by extracting the stain with 100 mmol/l cetylpyridinium chloride (Fisher Scientific, USA) for 3 h, and the absorbance was measured at 540 nm.

For the proteomics analyses, VICs (*n* = 4 donors) from duplicate wells of the calcification assay were lysed with radioimmunoprecipitation assay buffer supplemented with 1% protease inhibitor cocktail (Roche, Switzerland). Protein content was quantified using the BCA kit (Thermo Fisher Scientific, USA). For proteolysis, we used the iST Sample Preparation Kit (PreOmics GmbH, Germany), according to the manufacturer's instructions, using 7.5 μg of protein per sample. Eluted peptides were vacuum dried and resuspended in 40 μl of 5% acetonitrile (OptimaTM LC–MS grade; Thermo Fisher Scientific, USA) and 5% formic acid (Sigma–Aldrich, USA) prepared in OptimaTM LC–MS grade water (Thermo Fisher Scientific) for subsequent analysis.

### RNA analysis

VICs were lysed in Trizol (Life Technologies, USA), and RNA was isolated. Complementary DNA synthesis was conducted with the qScript complementary DNA Synthesis Kit (QuantaBio, USA). mRNA expression was assessed by TaqMan real-time PCR (Life Technologies, USA) with the following probes: Hs02758991_g1 (human GAPDH); Hs00154192_m1 (human BMP-2); Hs00985639_m1 (human IL-6). Expression levels were normalized to GAPDH. Results were quantified using the ^ΔΔ^CT method.

### Immunohistochemistry and immunofluorescence

AV samples were embedded in optimal cutting temperature compound (VWR, USA) and cut into 7 μm cryosections. All sections were fixed in 4% paraformaldehyde prior to staining. We performed blocking with the applicable serum followed by primary antibody incubation (Table S3).

Biotin-labeled secondary antibodies (1:200; Vector Laboratories, USA) and Alexa Fluor 488 and 594 antibodies (1:200; Life Technologies, USA) were used. Sections were embedded in 4,6-diamidino-2-phenylindole mounting medium (Vector Laboratories). We used streptavidin-labeled horseradish peroxidase solution (Dako, USA) and 3-amino-9-ethylcarbazole (Dako, USA) solution for staining development. Immunohistochemistry samples were examined with an Eclipse 80i microscope (Nikon, USA). Immunofluorescence sections were analyzed with a Nikon A1 confocal microscope (Nikon, USA).

### Oil red O staining

We used an Oil red O Stain Kit (ab150678; Abcam, UK) to visualize neutral lipid droplets on AV sections. Cryosections were placed in propylene glycol for 2 min, oil red O solution for 1 h, 85% propylene glycol for 1 min, and then rinsed with distilled water. The samples were then stained for nuclei with Mayer's hematoxylin.

### FACS

Freshly isolated VICs were collected by centrifugation and evenly split into FACS tubes (5 ml). Briefly, cells were centrifuged at 500*g* at 4 °C for 5 min, and the cell pellets were resuspended in 500 μl of PBS and 1 μl of Live and Dead stain (Zombie Aqua Fixable Viability Kit; BioLegend, USA). The samples were incubated at 4 °C for 20 min, protected from light, and then washed with FACS buffer (BD Biosciences, USA) and centrifuged at 500*g* at 4 °C for 5 min. The samples were blocked with 10% normal goat serum in PBS and incubated for 15 min at room temperature. The samples then were centrifuged at 500*g* at 4 °C for 5 min and incubated with CD68 (Novus Biologicals, USA; 600–985APC) diluted in FACS buffer (1:40) and supplemented with 10% normal goat serum for 20 min at 4 °C followed by washing with FACS buffer and centrifugation at 500*g* at 4 °C for 5 min. After that, samples were fixed with 2% paraformaldehyde (Biotium, USA) for 10 min at room temperature and washed with FACS buffer. The samples were incubated with 500 μl of Foxp3 fixation/permeabilization working solution (Ebioscience, USA) overnight at 4 °C. The samples then were washed with 1× permeabilization buffer (Ebioscience, USA) and centrifuged at 500*g* at 4 °C for 5 min. The cell pellets were resuspended in 200 μl of 1× permeabilization buffer and αSMA (R&D Systems, USA; IC1420S-025-APC-Cy7), ApoJ (Novus Biologicals, USA; 89884-APC), and ApoD (Novus Biologicals, USA; 42526) (1:40 dilution) and incubated for 45 min at room temperature. The cells were washed with FACS buffer twice at 500*g* at 4 °C for 5 min and incubated with ApoD followed by secondary antibody-PE (Novus Biologicals, USA; 7590) for 45 min at room temperature. The samples were all washed again with FACS buffer at 500*g* at 4 °C for 5 min, three times, and resuspended in 300 μl of FACS buffer for analysis using the BD FACSMelody cell sorter on 3 × 10^5^ cells per sample. Data were analyzed using FlowJo software (BD, USA), whereas fluorescent signals were gated to the control sample containing only the live/dead stain.

### Human AV proteomics and RNA-Seq data sets

AV leaflet data-dependent acquisition (DDA) mass spectrometric data corresponding to nine donor leaflets dissected into NF/NC, F, and C CAVD tissues from our previous study (25) were reanalyzed using an updated Proteome Discoverer software (version 2.2; Thermo Scientific) that performs peptide peak and chromatographic alignment for label-free quantification (see MS data analysis). RNA-Seq data from three donor AV leaflets dissected into NF/NC, F, and C CAVD tissues are from our previous study (25). We reanalyzed normalized read counts using fold-change values between CAVD tissues to determine the CAVD tissue–specific changes to apolipoprotein transcripts. Each data point is the average read counts per gene and per CAVD tissue of *n* = 3 donors.

### MS

#### Apolipoprotein peptide library and PRM

Peptide samples corresponding to five donor valves were analyzed with the high-resolution/accuracy Q Exactive mass spectrometer fronted with a Nanospray FLEX ion source, coupled to an Easy-nLC1000 HPLC pump (Thermo Scientific). The peptides were subjected to a dual-column setup: an Acclaim PepMap RSLC C18 trap column, 75 μm × 20 mm and an Acclaim PepMap RSLC C18 analytical column 75 μm × 250 mm (Thermo Scientific). We first analyzed the newly prepared AV peptide samples using unbiased peptide sampling (DDA) in order to confirm apolipoprotein peptides. The DDA analytical gradient was run at 300 nl/min from 5 to 18% solvent B (acetonitrile/0.1% formic acid) for 120 min, followed by 4 min of 95% solvent B. Solvent A was 0.1% formic acid. The instrument was set to 140 K resolution, and the top 10 precursor ions (within a scan range of 380–2000 *m*/*z*) were subjected to high-energy collision-induced dissociation (collision energy 25% [±2.5%], isolation width 1.6 *m*/*z*, dynamic exclusion enabled [20 s], and resolution set to 17.5 K).

A peptide library for 14 apolipoproteins was generated using apolipoprotein peptides observed from DDA experiments mentioned previously and plasma HDL samples analyzed in previous studies (25, 27) (Supporting Information 1 and 6). Stable isotope–labeled peptides (Supporting Information 6*A*; New England Peptide, USA; quantified by amino acid) with a final injection amount of 4 fmol apoA-I, 4 fmol apoE, and 0.4 fmol apo(a) were chosen as three independent universal normalization methods making this tier 2 assay; however, not as highly multiplexed for typical tier 2 assays. ApoA-I and apoE peptides ionize well as determined by our previous studies (27); the apo(a) peptide was added to independently verify trends of its endogenous peptide. We were able to monitor two to three peptides for all but one apolipoprotein, apoM (a single peptide), for a total of 32 peptides (Supporting Information 6*B*). The PRM analytical gradient was condensed to 35 min (Supporting Information 6*B*). The peptides were divided into three acquisition sets to increase sampling time per peptide by limiting retention time (RT) overlap as much as possible (Supporting Information 6*B*). The apoA-I standard was included in each PRM acquisition set; apoE in PRM acquisition sets 1 and 2; and apo(a) in acquisition set 2 in order to quantify their respective endogenous peptides, and corroborate relative quantification by apoA-I (Supporting Information 6*B*). We included a full MS1 scan (resolution, *R* = 140 K; scan range of 380–2000 *m*/*z*) per cycle in order to collect MS1 and MS/MS (PRM) data for subsequent quantification. Dissociation was set as in the DDA experiment, but the isolation window was widened slightly to 2 *m*/*z*.

### Data analysis

Since PRM data comprise full MS/MS scans, we were able to analyze them using Proteome Discover (version 2.1). The resulting PD 2.1 protein and peptide output files (.msf files) were imported into Skyline (34) as a reference library. The PRM files themselves (.RAW files) were imported into Skyline for quantification of targeted peptides using the normalized area under the curve of the extracted ion chromatograms of the precursor and three to four of the most intense PRM ions per peptide. All peaks were manually validated, and integration boundaries for low abundant signals were adjusted to exclude background. We confirmed the identities and RTs of the monitored apolipoproteins by analyzing four previously studied HDL size fractions, alpha0, alpha1, alpha2, and alpha3 (Supporting Information 1) (27). In order to verify that this quantification strategy can detect changes in abundance of any given apolipoprotein across the valve tissue, we normalized their abundances to those of the stable isotope standards.

#### VIC lysate MS

VIC peptide samples were analyzed on the Orbitrap Fusion Lumos mass spectrometer fronted with an EASY-Spray Source (heated at 45 °C) and coupled to an Easy-nLC1000 HPLC pump (Thermo Fisher Scientific). Peptides were subjected to a dual-column setup: an Acclaim PepMap RSLC C18 trap analytical column, 75 μm × 20 mm (precolumn) and an EASY-Spray LC column, 75 μm × 250 mm (Thermo Fisher Scientific). The analytical gradient was run at 300 nl/min from 5 to 21% solvent B (acetonitrile/0.1% formic acid) for 75 min, 21 to 30% solvent B for 15 min, followed by 10 min of 95% solvent B. Solvent A was water/0.1% formic acid. The acetonitrile and water were of MS grade. The Orbitrap analyzer was set to 120 K resolution, and the top N precursor ions in 3 s cycle time within a scan range of 375 to 1500 m/z (60 s dynamic exclusion enabled) were subjected to collision-induced dissociation (collision energy, 30%; isolation window, 1.6 *m*/*z*; automatic gain control target, 1.0 e4). The ion trap analyzer was set to a rapid scan rate for peptide sequencing (MS/MS).

### MS data analysis

Both human AV and VIC MS/MS data were queried against the Human UniProt database (downloaded November, 2018, *n* = 155,133 entries) using the HT-SEQUEST search algorithm *via* Proteome Discoverer, version 2.2 (PD2.2). Oxidation of methionine was set as a variable modification, and carbamidomethyl of cysteine was set as a fixed modification. The enzyme was set to trypsin (full), with a maximum of four missed cleavages, using a 10 ppm precursor tolerance window and a 0.02 Da fragment tolerance window. In order to quantify peptide precursors detected in the MS1 but not sequenced from sample to sample, we enabled the Feature Mapper node. Chromatographic alignment was done with a maximum RT shift of 10 min and a mass tolerance of 10 ppm. Feature linking and mapping settings were as follows: RT tolerance minimum of 0 min, mass tolerance of 10 ppm, and signal-to-noise minimum of 5. Total peptide amount was used for normalization. Peptides were filtered based on a 1% false discovery rate (FDR) as determined using the reverse decoy database search strategy (35, 36).

### AV tissue and VIC proteomics data analysis

For principal component analysis and heat maps, we filtered out genes or proteins by their *q*-value of multiple group comparisons. The *q*-value is the proportion of the rejected null hypotheses, which are erroneously rejected and is a type of FDR (37). The *q*-value was calculated using Qlucore, and its thresholds were indicated in the legend to the figures.

#### AV tissue proteomics

Proteins with ≥3 unique peptides were included in the analysis. The multigroup analysis compared proteins from the three CAVD tissues with a *q*-value of ≤0.3. Variances because of sample processing batch effects were accounted for in the multigroup comparison (Qlucore Omics Explorer). To plot the relative abundances of individual proteins, the normalized PD2.2 protein abundances were used. Statistical analyses of these data were performed using GraphPad PRISM (GraphPad Software, USA) and SPSS (SPSS, Inc, Chicago, IL, USA). *p* < 0.05 was considered statistically significant.

#### VIC *in vitro* proteomics

Proteins with ≥2 unique peptides were included in the analysis. The normalized PD2.2 protein abundances were used. For the statistical analysis, we used in-house scripts of R, version 3.1 (http://www.R-project.org/) and Python, version 3.8.1. (http://www.python.org). Enriched proteins were defined by a *q*-value of ≤0.1 between NM, PM, and PM + ApoC-III. For the protein modules and pathway analyses (Fig. 6, *A*–*B*; Supporting Information 3 and 4, *A*–*B*) differentially enriched proteins with a *q*-value of ≤0.1 were considered with a fold change >1 between apoC-III and NM or PM, respectively.

#### PPI networks, pathway enrichment analysis, and pathway maps

To build the protein interaction subnetworks, proteins enriched in apoC-III *versus* NM or PM were mapped to the global human PPI network described (38), using NetworkX, version 2.4 (39). Using ConsensusPathDB (40), the protein sets corresponding to an enrichment in ApoC-III *versus* NM or PM were tested for enrichment by a hypergeometric test and adjusted for multiple comparisons using the Benjamini–Hochberg method for controlling FDR. For the pathway enrichment analysis (pathway data retrieved from http://consensuspathdb.org/ in July 2020), the canonical pathways from KEGG, BioCarta, and Reactome were considered (41). Pathways with a two-sided hypergeometric test *p*-value <0.05 (corresponding to FDR <0.15) are considered as significantly enriched. The pathway networks consist of pathways as the nodes and the shared genes between pathways as the edges. Edge weight (thickness) corresponds to the gene overlap between pairs of pathways measured by the Jaccard index J, which is defined as

$$J\;=\;\frac{s_{A}\cap s_{B}}{s_{A}\cup s_{B}}$$

where *s*_*A*_ and *s*_*B*_ are the set of proteins detected in proteomics that belong to pathway *A* and pathway *B*, respectively. Edges with a Jaccard index <0.1 were discarded in the visualization for clarity. The network visualizations were made using Gephi, version 0.9.2 (42). Pathway modules in the pathway maps were determined using the modularity optimization functionality within Gephi, version 0.9.2.

#### Interactome-based association analysis

We measured the network-based distance to quantify the association between the pathways enriched after apoC-III treatment and CAVD-related and vascular calcification–related pathways and proteins. We used the closest distance metric *d*_c_ as defined in (43) such that

$$d_{c}=\frac{1}{\left\|S\right\|}\sum_{s\text{∈}S}\begin{array}{l} \min\;d\left(s,t\right),\\ t\text{∈}T \end{array}$$

where *S* is the set of source genes (apoC-III pathway genes), *T* is the set of target genes (CAVD-related pathway genes and vascular calcification–related genes), and *d*(*s*, *t*) is the network distance measured as the number of edges between nodes *s* and *t* in *S* and *T*, respectively, in the PPI network described previously. The vascular calcification–related genes, in particular, the sets of calcificasome and inflammasome genes, were taken from (29), and the CAVD-related pathways were taken from (25). The genes belonging to CAVD-related pathways were extracted from the ConsensusPathDB. To assess the statistical significance of the measured distance between apoC-III pathways and CAVD and calcification-related genes and pathways, we followed a degree-preserving randomization approach and determined empirical *p*-values as described previously (25) by performing 200 randomizations.

### Additional statistical analyses

Multigroup comparisons were performed using ANOVA with a Bonferroni post-test for parametric data and Kruskal–Wallis test with Dunn's post-test for nonparametric data. PRM ion intensities of the apolipoprotein peptides were sum normalized between CAVD tissues. Statistical analyses were conducted on the averaged sum normalized intensities of one to three peptides per apolipoprotein to represent one signal per donor. Only apolipoproteins with quantifiable PRM ion intensities for all five donors in the PRM study were analyzed in the tissue proteomics. Tissue-type enrichment was defined as statistically significant difference in sum-normalized abundance in any of the three tissue types (NF/NC, F, and C) *versus* any other types of tissue. Statistical analyses were performed using GraphPad PRISM. *p* < 0.05 was considered statistically significant.

## Data availability

All MS and resulting search data (the reanalyzed AV leaflet data sets and the newly acquired AV PRM and VIC calcification data sets) have been deposited to the ProteomeXchange Consortium *via* the PRIDE partner repository (44) with the data set identifier PXD021858.

## Conflict of interest

The authors declare that they have no conflicts of interest with the contents of this article.

## Supporting information

JBC_JBC/2020/015700_10

JBC_JBC/2020/015700_8

JBC_JBC/2020/015700_9
