# Supplementary material for: ApoC-III is a novel inducer of calcification in human aortic valves
Source: J Biol Chem. 2021 Jan 6;296:100193. doi: 10.1074/jbc.RA120.015700 (PMC7948477; doi:10.1074/jbc.RA120.015700)
Supplement: JBC_JBC/2020/015700_8 [file mmc2.pdf]

ApoC-III is a novel inducer of calcification in human aortic valves

Florian Schlotter, MD*[1], Renata C. C. de Freitas, MSc*[1], Maximillian A. Rogers, PhD[1], Mark C. Blaser, PhD[1], Pin-Jou Wu, MS[1], Hideyuki Higashi, MS[1], Arda Halu, PhD[1,2], Farwah Iqbal, PhD[3], Allison B. Andraski, PhD[4], Cayla N. Rodia, PhD[5], Shiori Kuraoka, MSc[1], Jennifer R. Wen, BS[1], Michael Creager, BS[1], Tan Pham, BS[1], Joshua D. Hutcheson, PhD[6], Simon C. Body, MD, MPH[7], Alison B. Kohan, PhD[8], Frank M. Sacks, MD[4], Masanori Aikawa, MD, PhD[1,3], Sasha A. Singh, PhD[1], Elena Aikawa, MD, PhD[1,3,9]

[1] Center for Interdisciplinary Cardiovascular Sciences, Division of Cardiovascular Medicine, Department of Medicine, Brigham and Women's Hospital, Harvard Medical School, Boston, MA, USA

[2] Channing Division of Network Medicine, Brigham and Women's Hospital, Harvard Medical School, Boston, MA, USA

[3] Center for Excellence in Vascular Biology, Division of Cardiovascular Medicine, Department of Medicine, Brigham and Women's Hospital, Harvard Medical School, Boston, MA, USA

[4] Department of Nutrition and Department of Molecular Metabolism, Harvard T.H. Chan School of Public Health, Boston, MA, USA

[5] Department of Nutritional Sciences, University of Connecticut, Storrs, CT, USA

[6] Department of Biomedical Engineering, Florida International University, Miami, FL, USA

[7] Department of Anesthesiology, Boston University School of Medicine, Boston, MA, USA

[8] Department of Medicine, Division of Endocrinology and Metabolism, University of Pittsburgh, PA, USA

[9] Department of Human Pathology, Sechenov First Moscow State Medical University, Moscow, Russia

Short title: Role of ApoC-III in calcific aortic valve disease

Corresponding author:
Elena Aikawa, M.D., PhD
Center for Interdisciplinary Cardiovascular Sciences
Brigham and Women's Hospital, Harvard Medical School
3 Blackfan St, 17th Floor
Boston, MA 02115
Phone: 617 730-7755
Email: eaikawa@bwh.harvard.edu
Twitter handle: @CICSnews

Supporting Information 1

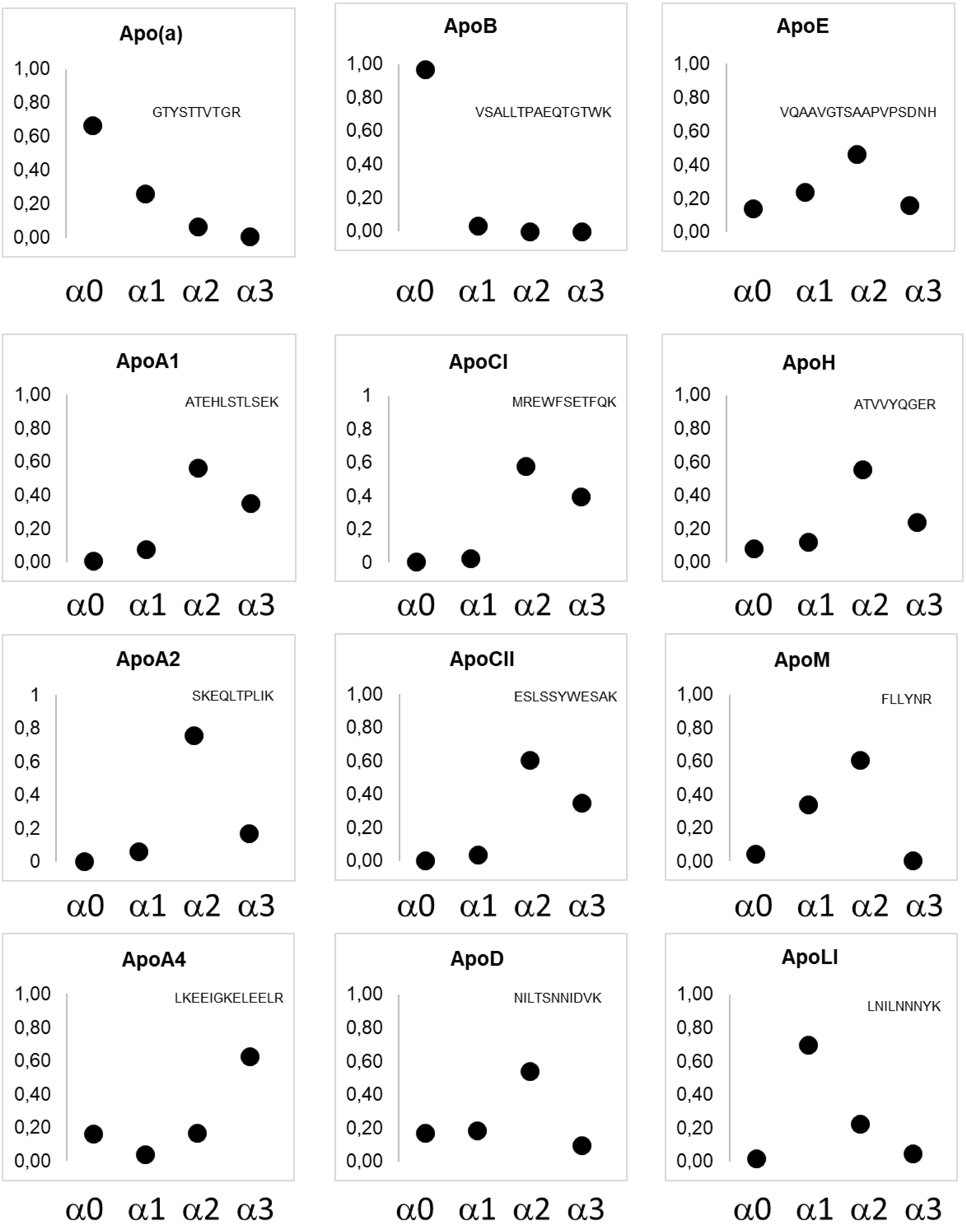

Supporting Information 2

Gating Strategy

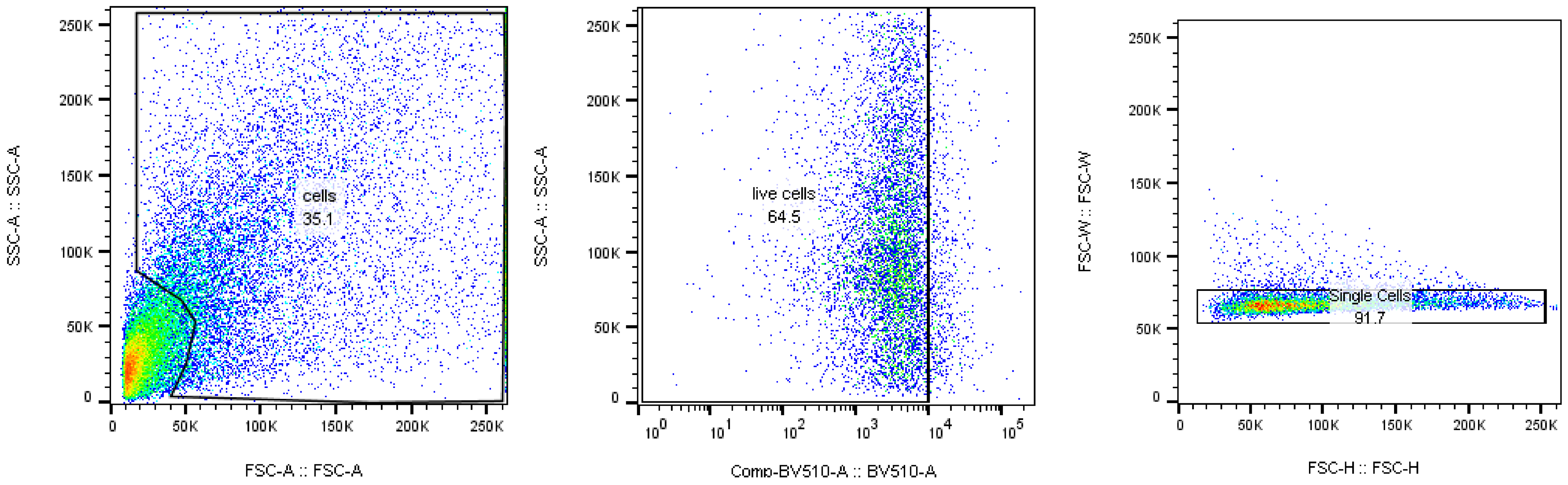

CD68 (APC) + ApoD (PE)

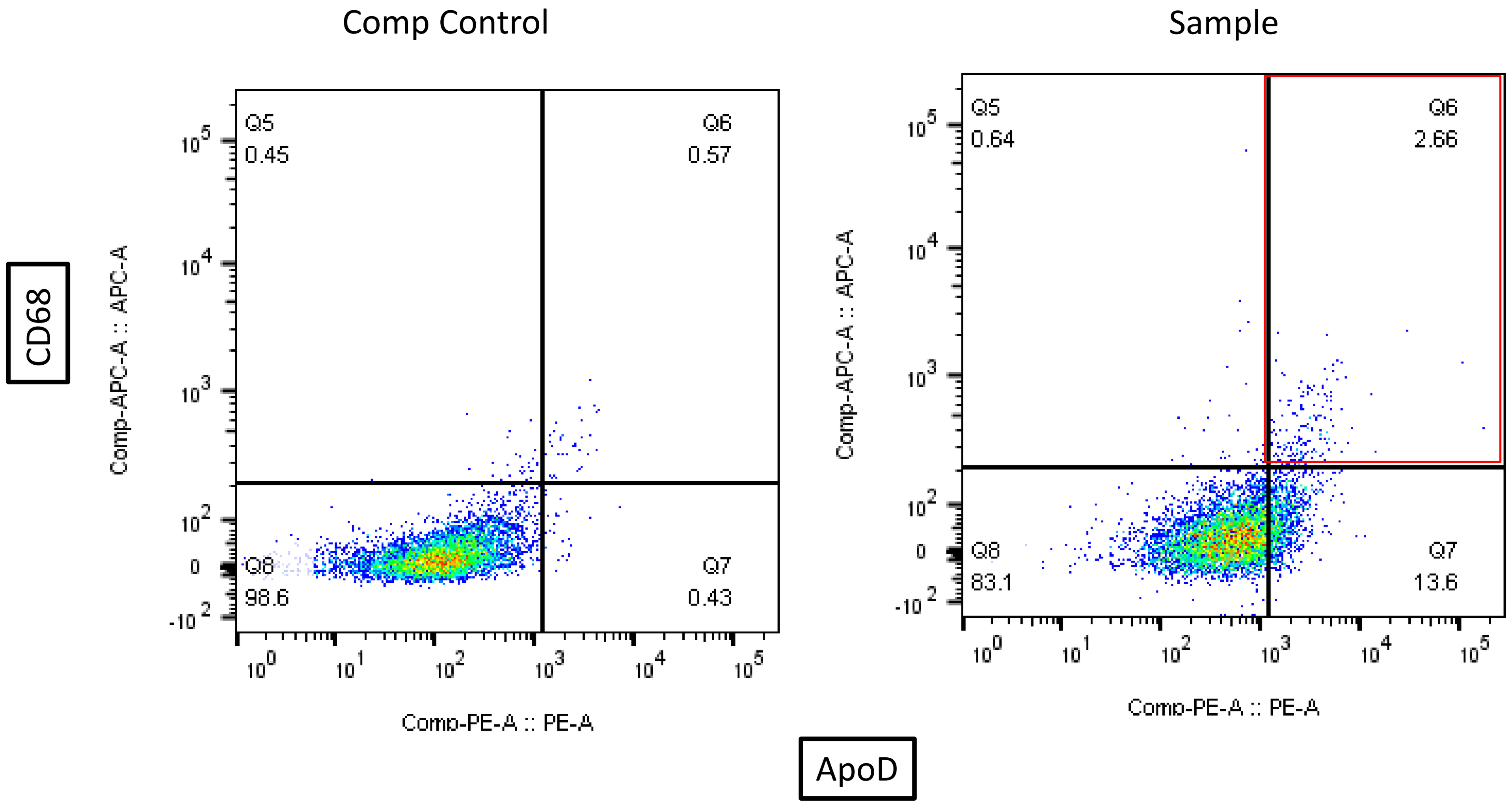

APOJ (APC) + aSMA (APC-cy7)

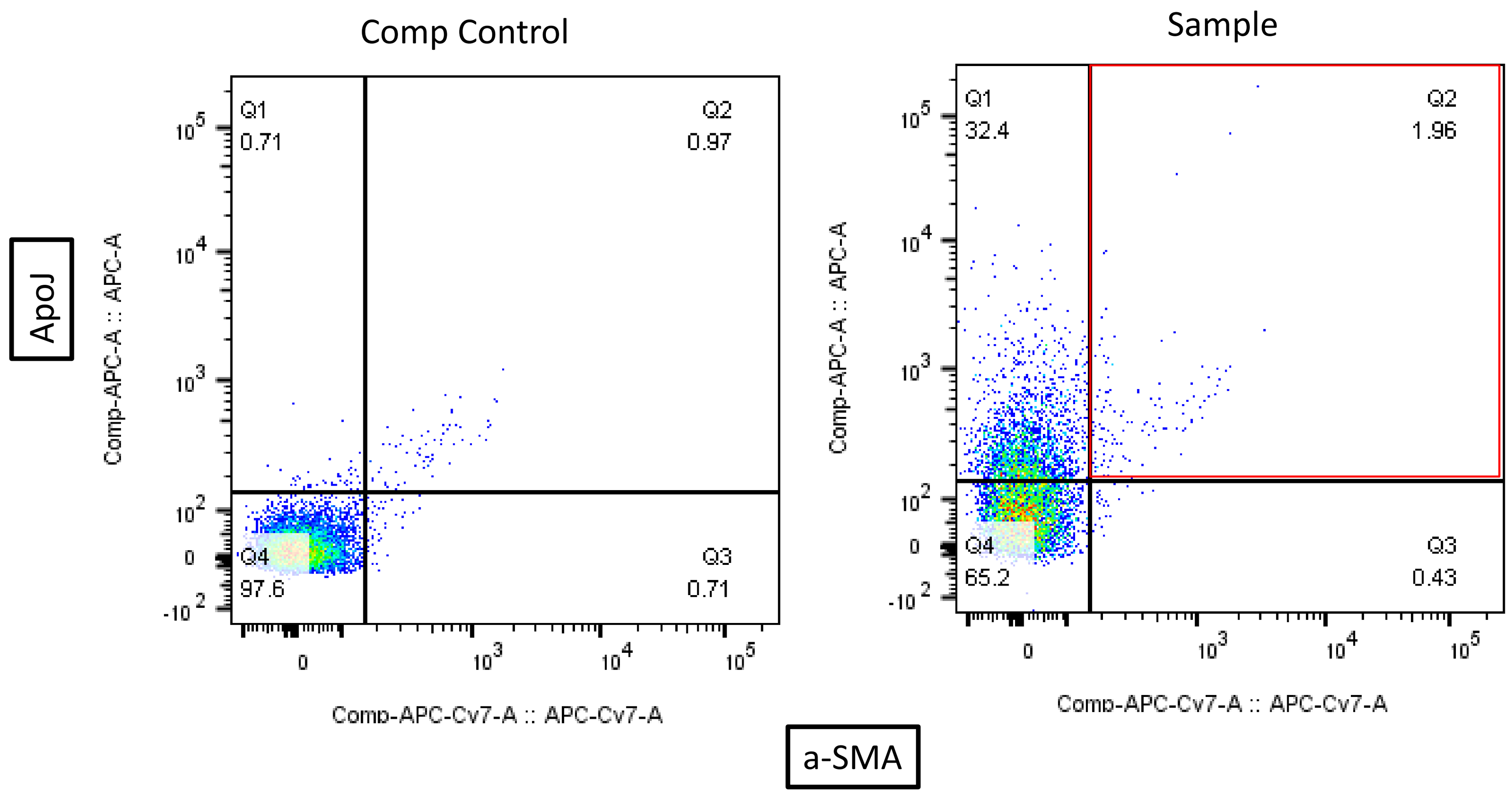

Supporting Information 3

Enriched in apoC-III vs. NM protein module

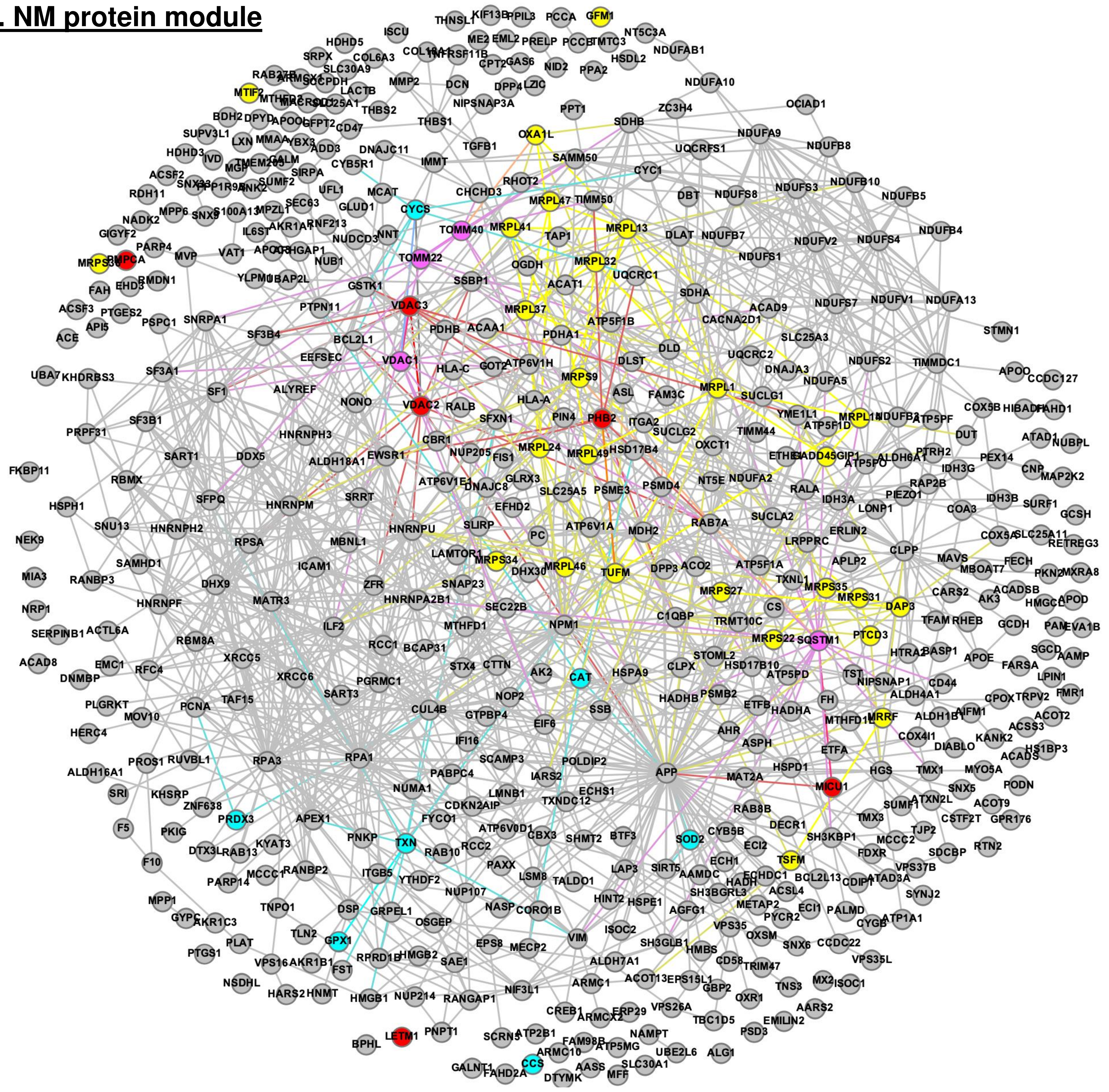

Enriched in apoC-III vs. PM protein module

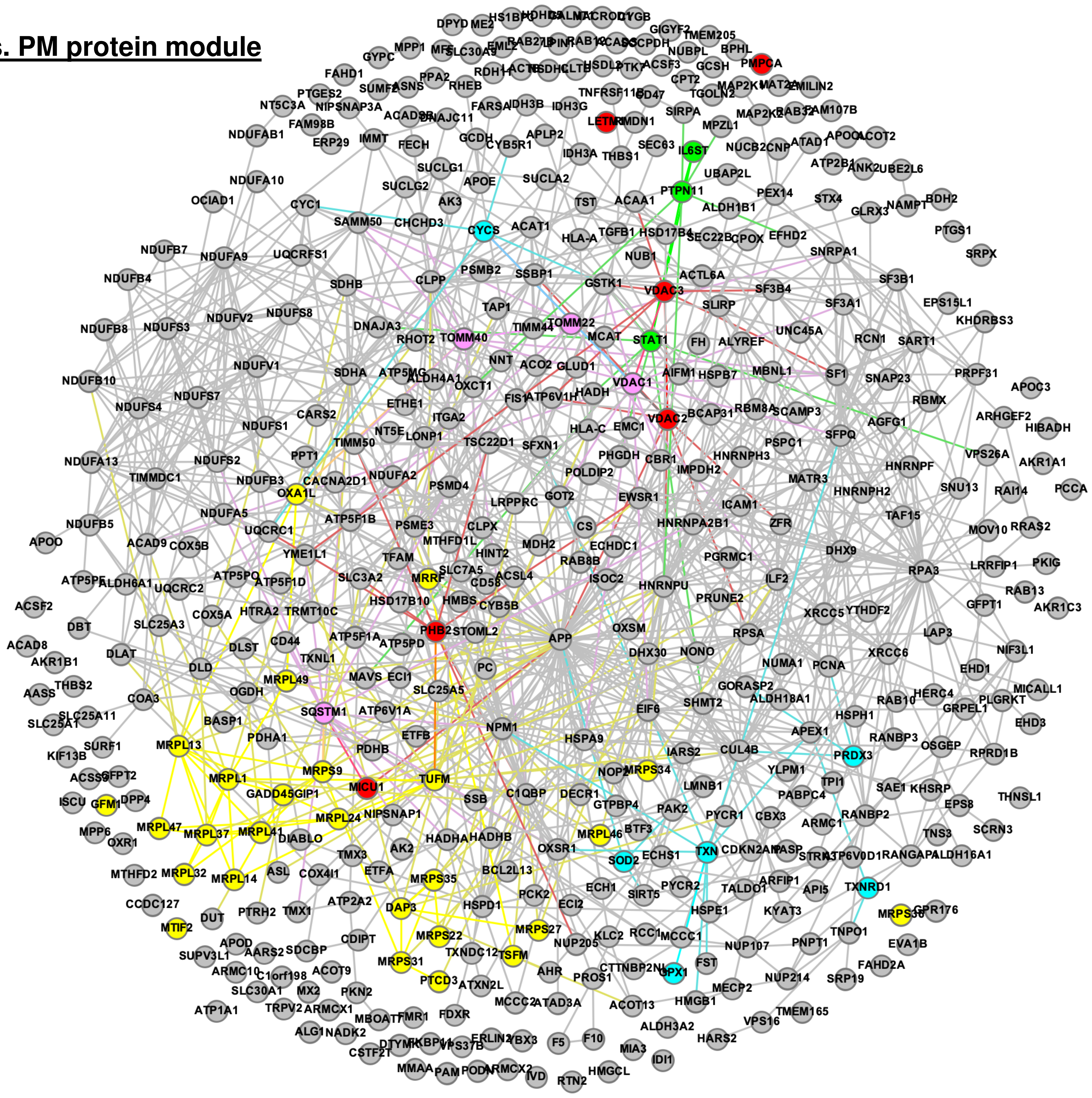

Supporting Information 4A

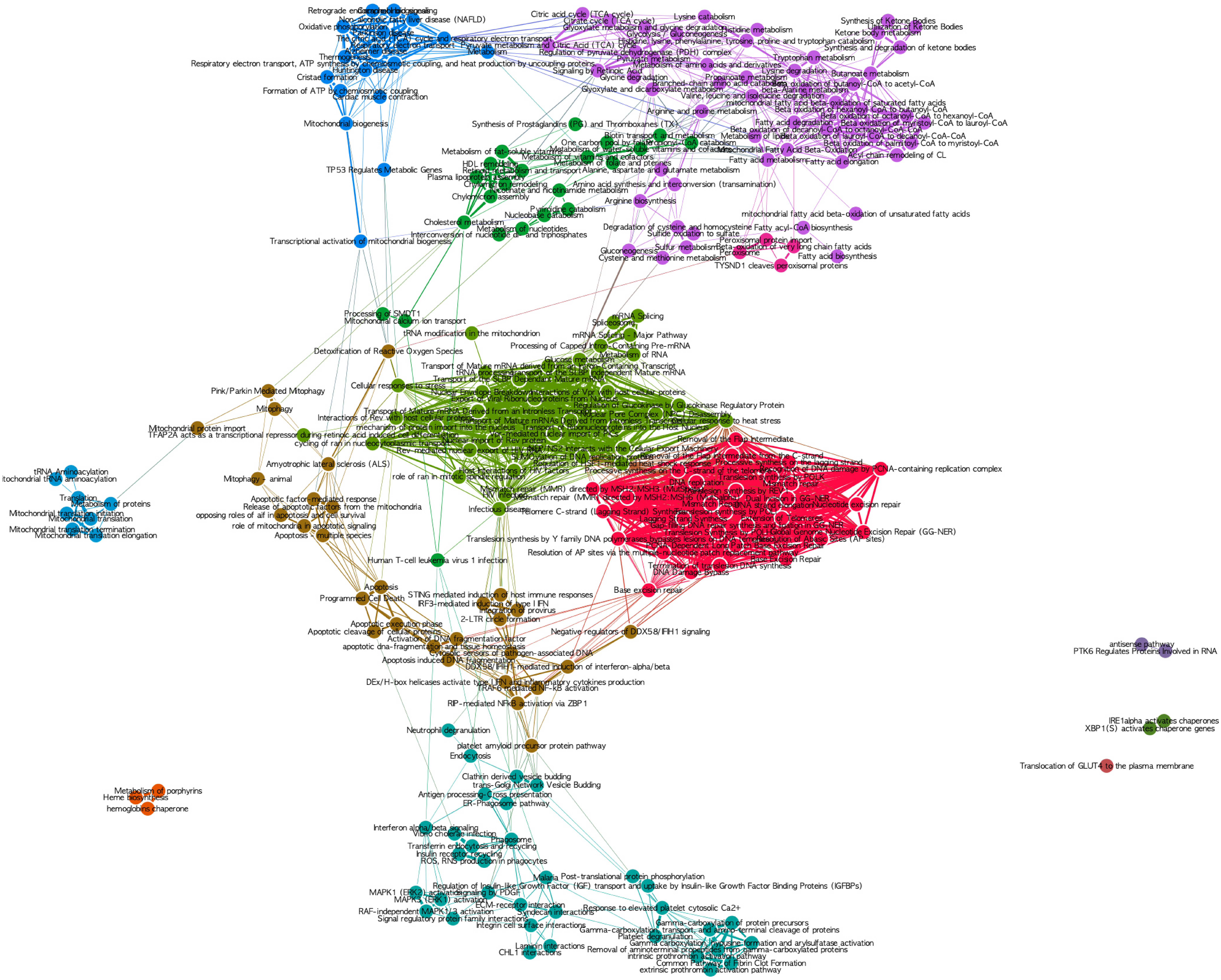

Supporting Information 4B

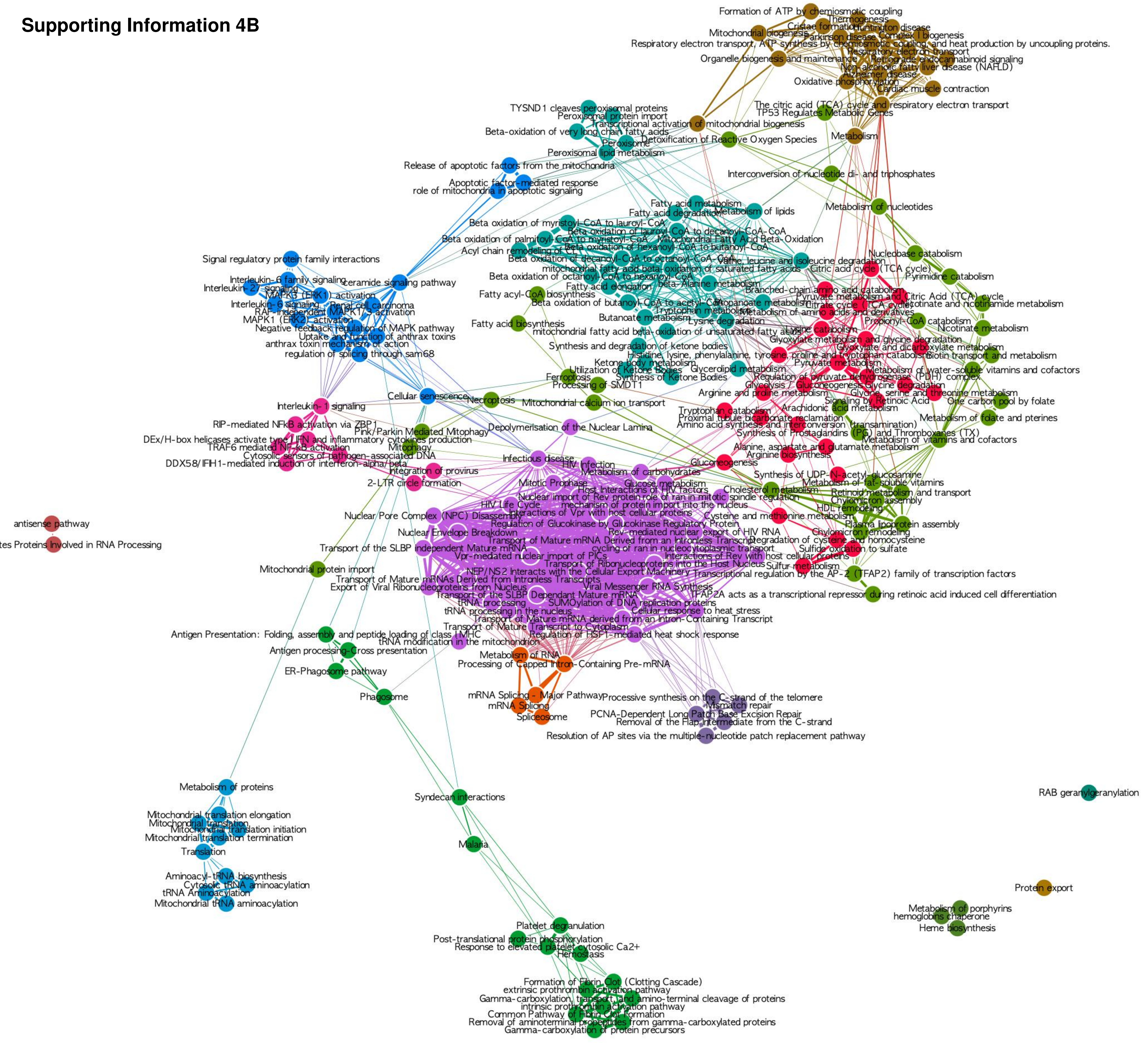

Supporting Information 5

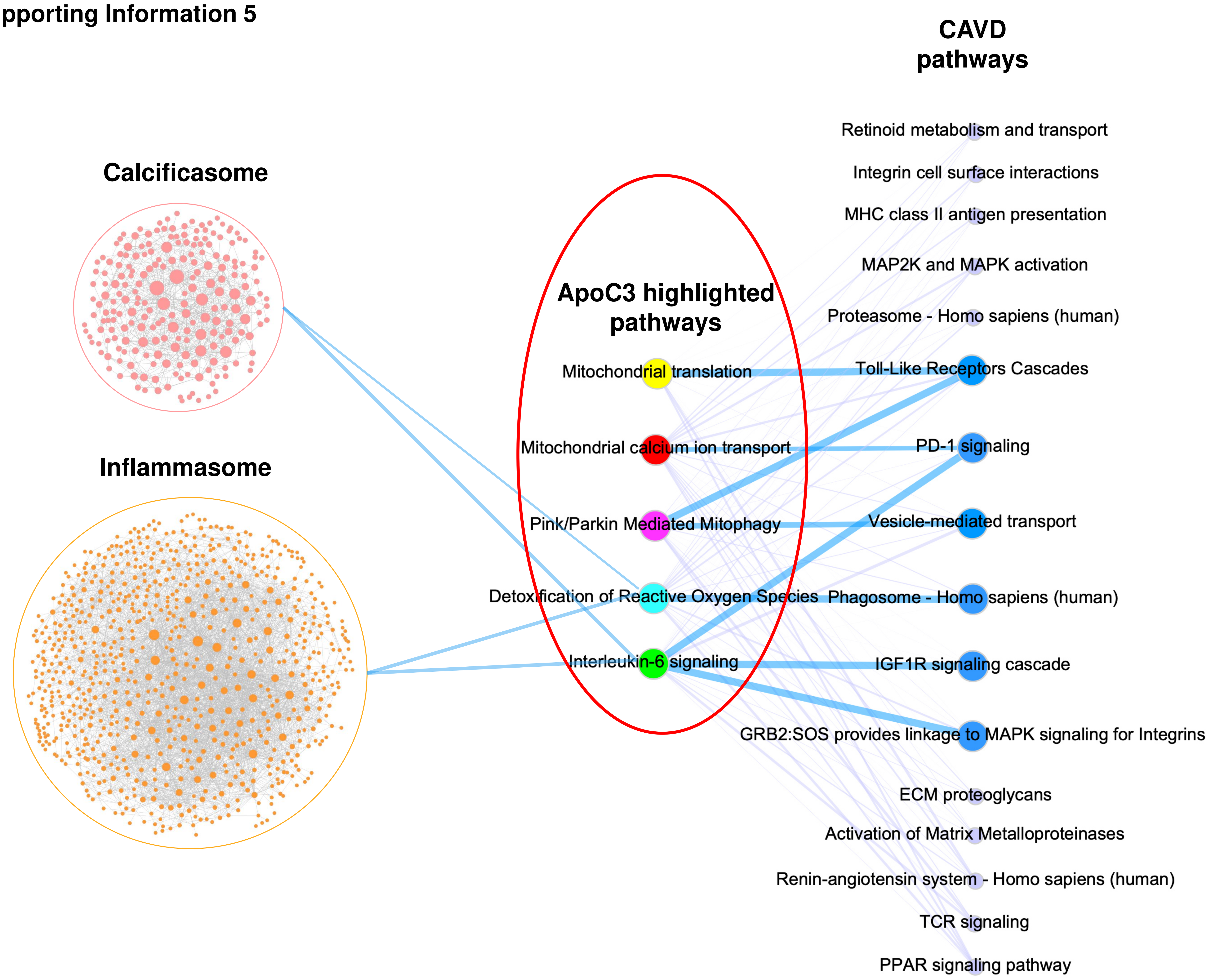

Supporting Information 6

A)

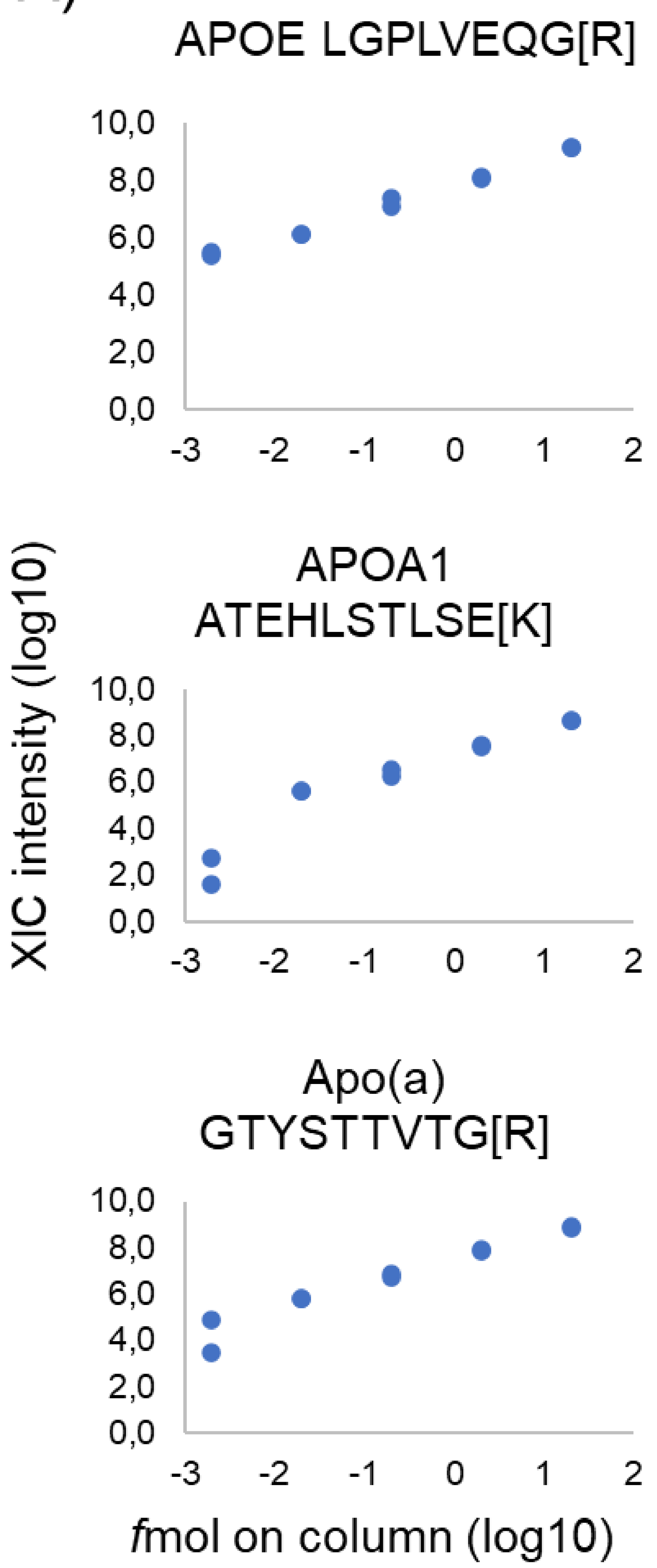

B)

	Mass [m/z]	CS [z]	Start [min]	End [min]	SEQUENCE_APOLIPOPROTEIN	Obser
PRM set 1	384,212	3	6	9	KATVVYQGER_APOH	yes
	405,8787	3	8	11	ATEHLSTLSEK_APOA1	yes
	408,55	3	8	11	**ATEHLSTLSEK[label]_APOA1**	no
	644,3362	2	8	12	TQVNTQAEQLR_APOA4	yes
	438,5772	2	9	12	AKLEEQAQQIR_APOE	yes
	511,7669	2	9	12	ATVVYQGER_APOH	yes
	484,7798	2	12	15	LGPLVEQGR_APOE	yes
	489,78	2	12	15	**LGPLVEQGR[label]_APOE**	yes
	644,8226	2	15	19	ELDESLQVAER_APOJ	yes
	858,9292	2	16	19	DALSSVQESQVAQQAR_APOC3	yes
	413,2345	2	17	20	FLLYNR_APOM	yes
	801,4303	2	20	23	VSALLTPAEQTGTWK_APOB	yes
	Mass [m/z]	CS [z]	Start [min]	End [min]	SEQUENCE_APOLIPOPROTEIN	
PRM set 2	496,7213	2	6	12	TDASDVKP[C]_APOH	yes
	405,8787	3	8	11	ATEHLSTLSEK_APOA1	yes
	408,55	3	8	11	**ATEHLSTLSEK[label]_APOA1**	no
	521,7618	2	9	12	GTYSTTVTGR_apo(a)	yes
	526,77	2	9	12	**GTYSTTVTGR[label]_apo(a)**	yes
	436,2534	3	9,5	12,5	VLNQELR_APOD	yes
	477,7337	2	10	15	FMETVAEK_CLUS(APOJ)	yes
	815,8996	2	12	15	VTEPISAESGEQVER_APOL1	yes
	471,2869	2	15	18	EQLTPLIK_APOA2	yes
	529,2981	3	15	18	LKEEIGKELEELR_APOA4	yes
	509,2489	3	19	23	AEPLAFTFSHDYK_APOB	yes
	981,4479	2	25	35	TPEYYPNAGLIMNY[C]R_LPA	no
	Mass [m/z]	CS [z]	Start [min]	End [min]	SEQUENCE_APOLIPOPROTEIN	
PRM set 3	473,248	2	7	10	VAQELEEK_APOL1	yes
	486,7535	2	8	11	SPELQAEAK_APOA2	yes
	405,8787	3	8	11	ATEHLSTLSEK_APOA1	yes
	408,55	3	8	11	**ATEHLSTLSEK[label]_APOA1**	no
	519,2667	2	9	12	TAAQNLYEK_APOC2	yes
	386,2361	3	11	14	SKEQLTPLIK_APOA2	yes
	810,9025	2	11	14	VQAAVGTSAAPVPSDNH_APOE	yes
	526,7484	2	12	15	EFGNTLEDK_APOC1	yes
	415,9065	3	14	19	TLLSNLEEAKK_CLUS(APOJ)	no
	553,3037	2	15	18	LNILNNNYK_APOL1	yes
	615,8381	2	16	19	NILTSNNIDVK_APOD	yes
	449,7189	2	16	19	DYWSTVK_APOC3	yes
	496,9029	3	18	23	MREWFSETFQK_APOC1	yes
	643,7986	2	20	24	ESLSSYWESAK_APOC2	yes
	806,8963	2	30	35	LLDNWDSVTSTFSK_APOA1	yes

Supporting Information Legend

Supporting Information 1: Targeted PRM mass spectrometry for the selected apolipoprotein peptides across four high-density lipoprotein (HDL) size fractions from large to small (alpha0, alpha1, alpha2, alpha3).

Supporting Information 2: Fluorescence-activated cell sorting of freshly isolated primary human valve cells: top panel: gating strategy; middle panel: CD68 and apoD expression; lower panel: alpha-smooth muscle actin (αSMA) and apoJ expression

Supporting Information 3:

A) Proteins with $q \leq 0.1$ between NM, PM and PM + apoC-III increased in PM + apoC-III vs. NM (n=4 donors, day7 and day14 data).

B) Proteins with $q \leq 0.1$ between NM, PM and PM + apoC-III increased in PM + apoC-III vs. PM (n=4 donors, day7 and day14 data).

Supporting Information 4: ApoC-III Functional Analyses

A) The network of significantly enriched pathways (two-sided hypergeometric test p-value <0.05) in the differential proteome of apoC-III vs. NM based on unbiased community detection.

B) The network of significantly enriched pathways (two-sided hypergeometric test p-value <0.05) in the differential proteome of apoC-III vs. PM based on unbiased community detection.

Supporting Information 5: Interactome-based association analysis

Network association of select apoC-III-associated pathways (middle column, derived from the data in Fig. 6A/B) with previously published inflammation and calcification modules (left) and calcific aortic valve disease (CAVD)-related pathways (right column; Experimental procedures for references). Edge thickness represents degree of association.

Supporting Information 6:

A) Standard curve with three stable isotope labeled peptides fully labeled (13C, 15N) at the Arg or Lys residues. Valve peptide samples were mixed with standards to a final concentration of 4 *f*mol apoA-I, 4 *f*mol apoE, and 0.4 *f*mol apo(a) on column. B) PRM inclusion list (peptides in bold represent the stable isotope labeled peptides). Start and end time represent the start and end time of the retention time window.

Supporting Table 1: CAVD tissue proteome (all data)

Supporting Table 2: Proteome data of apolipoprotein treated human VICs in cell culture

Table S3:

Protein	Supplier	Host species	Dilution
Lipoprotein (a)	abcam (MA, USA)	Goat polyclonal	1:50
Apolipoprotein A-I	abcam (MA, USA)	Rabbit monoclonal [EP1368Y]	1:100
Apolipoprotein A-II	abcam (MA, USA)	Mouse monoclonal	1:100
Apolipoprotein A-IV	abcam (MA, USA)	Mouse monoclonal [CL0468]	1:100
Apolipoprotein B	abcam (MA, USA)	Goat polyclonal	1:100
Apolipoprotein C-III	abcam (MA, USA)	Rabbit monoclonal [EP1372Y]	1:100
Apolipoprotein D	abcam (MA, USA)	Rabbit monoclonal [EPR2916]	1:100
Apolipoprotein E	abcam (MA, USA)	Mouse monoclonal [E6D7]	1:100
Apolipoprotein J	abcam (MA, USA)	Rabbit monoclonal [EPR2911]	1:400
Apolipoprotein M	abcam (MA, USA)	Goat polyclonal	1:100
